# Inclusivity and Engagement: The Canadian Spinal Cord Injury – Rehabilitation Association Welcomes You to the 10th National Spinal Cord Injury Conference

**DOI:** 10.46292/1945-5763-29.suppl.ii

**Published:** 2023-11-17

**Authors:** Kristin E. Musselman, B. Catharine Craven

At this year's 10th National Spinal Cord Injury Conference, the Canadian Spinal Cord Injury– Rehabilitation Association (CSCI-RA) is celebrating its emphasis on inclusivity and engagement, two constructs increasingly present in our collective consciousness. *Inclusion* is the practice of creating environments and situations where all people have equal access to opportunities, resources, and knowledge. Effective inclusion will, in turn, lead to meaningful engagement, which is achieved when people with lived experience are involved in decision-making processes related to clinical care, research, knowledge sharing, and community initiatives. The intent of the conference theme is not to highlight the benefits of inclusion and engagement in clinical and research environments; we already know that diverse and inclusive teams tend to be more creative, innovative, and relevant. Rather, the conference is an opportunity to consider how we can best support inclusion and engagement in current clinical practice, research endeavors, technology development, knowledge translation, and implementation initiatives.

There are numerous ways we can support inclusivity and engagement. One is to understand the challenges of minoritized individuals and communities. There is a growing understanding that many people see themselves as oppressed in some way, shape, or form. Disability justice advocates for use of an intersectionality lens that considers all the ways a person is oppressed when trying to understand a person's experience in the rehabilitation sector or health system. An intersectionality lens assumes that everyone has their own experiences of privilege and oppression in their personal, social, political, and economic contexts within their community, research, or clinical service program. Understanding one's own privilege and intersectional lens is an important step toward supporting minoritized communities.

The 10th National Spinal Cord Injury Conference will highlight the work of Dr. Stephanie Nixon, who developed the Coin Model of Privilege and Critical Allyship.^1^ The Coin Model provides a framework for self-reflection and action, encouraging us all to be aware of our privilege and consider how to use it to be an ally. Allyship involves supporting and advocating for minoritized groups whose voices are not being heard.^2^ In both rehabilitation practice and research, it involves reframing who is an expert and dismantling the power imbalances that traditionally exist in these settings.

One can support inclusivity and engagement through listening and amplifying the voices of persons with lived experience. Many spinal cord injury or disease (SCI/D) rehabilitation programs suffer from an ableism bias, which impedes disability justice, inclusion, and engagement. Inclusion of individuals living with SCI/D in the co-design of future programs and services is critical for fostering a more equitable healthcare environment. People living with SCI/D are experts by experience; their engagement in the development and implementation of health services and research projects will result in more impactful outcomes.

Researchers and healthcare providers are often unsure how to engage people with lived experience. The 10th National Spinal Cord Injury Conference is an opportunity for the SCI/D rehabilitation community to share resources and strategies for effective engagement through workshops and networking initiatives. Keynote speaker Barry Munro will share a historical perspective on engagement based on decades-long career-building partnerships and advocating for people living with SCI/D. In addition, this special issue of *Topics in Spinal Cord Injury Rehabilitation* shares articles modeling inclusion of people with lived experience in research and care initiatives, such as developing an evidence-based integrated care pathway and identifying key challenges to the implementation of activity-based therapy in Canada.

Sharing sociodemographic information, such as gender and sexual orientation, is essential to providing the highest quality healthcare for individuals with SCI/D. By acknowledging nonbinary genders and trans experiences, healthcare providers can become better at providing equitable services and inclusive care. To date, there is a paucity of literature describing how service delivery is influenced by gender and/or sexual orientation. Further, we also need to consider those with low income, for whom food, housing, and/or medical supply insecurity may be inadvertently impacting rehabilitation outcomes. As a community, we need to build trust to ensure people feel safe when sharing their personal information and trust that it will be used to optimize their outcomes.

In Canada, we have a history of leadership in the creation of large-scale datasets, such as the Rick Hansen SCI Registry^3^ and the Open Data Commons for Pre-Clinical SCI Research.^4^ Developing a transparent equity strategy will necessitate the creation of new datasets to support inclusivity and engagement in the SCI/D rehabilitation field. High quality, socially acceptable minimum datasets that include collection of sociodemographic variables, such as gender, sexual orientation, and race, will provide the data needed to effectively advocate for equitable services and inclusive care over time. We trust that you will use your time at the conference to reflect on your own privilege, oppression, and unconscious biases that may be influencing your perception or delivery of clinical care and research. Try to identify one to two learning opportunities for yourself beyond the conference and plan to meet with a small group of colleagues to discuss what you learned or how the conference influenced your perceptions of yourself.

Welcome to the 10th National Spinal Cord Injury Conference and to the multicultural and cosmopolitan city of Toronto! While visiting with us, we hope you have a chance to visit the Toronto Winter Village in the Distillery District, attend a live show within Toronto's Theatre District or take in a Raptor's or Leaf's game. Enjoy!
